# The Eudemonic Wellbeing of Volunteers in a Public Health Emergency: COVID-19 in China

**DOI:** 10.3389/fpsyg.2022.903147

**Published:** 2022-06-02

**Authors:** Juan Tang, Xiao-chen Li, Xi Zhang

**Affiliations:** ^1^Faculty of Hospitality and Tourism Management, Macau University of Science and Technology, Macau, Macau SAR, China; ^2^School of Innovation and Entrepreneurship, Guangdong Polytechnic Normal University, Guangzhou, China

**Keywords:** public health emergency, volunteer, eudemonic wellbeing, job involvement, empathy

## Abstract

With improvements in the public awareness regarding volunteer opportunities, more people are participating in social work, particularly during emergency events. The mental health of volunteers has been attracting more academic attention due to its increasing social significance. Drawing on the Theory of Planned Behavior, a qualitative interview was conducted to identify important attitudes, subjective norms, and perceived control factors guiding people’s volunteering behaviors in an emergency context. Then, a sequential quantitative survey was implemented based on the results of the qualitative study to explore the impact of the aforementioned factors and job involvement on eudemonic well-being. The moderating role of empathy in these relationships was also investigated in this nested design. The results indicate that behavioral attitudes, perceived control, and job involvement have significant positive effects on volunteers’ eudemonic well-being. A high perspective taking (cognitive empathy) of volunteers positively moderates the relationship between job involvement and eudemonic well-being, while high personal distress (affective empathy) buffers this relationship. The theoretical and practical implications of these findings are discussed in relation to emergency volunteer activities.

## Introduction

At the beginning of 2020, COVID-19 began to spread rapidly around the world and has been identified as a public health emergency by the World Health Organization (2020) (WHO), leading to the first-level response in many countries. With the rising public awareness regarding volunteer opportunities around the world (Crane, 2019), many volunteers offered their time and effort in this emergency response. In addition to the frontline doctors and nurses, volunteer teams were also deployed to perform emergency rescue services immediately. Being under the constant fear of exposure and uncertainty regarding the possibility of infection, the mental state of volunteers was intensively challenged in this severe public health event.

During and after the service, return of the volunteers back to their homes was identified as “not easy,” as some were required to be in “isolation” before re-entry (Curtin and Brown, 2019, p. 192). As such, pathological issues and psychological well-being (PWB) of volunteers were extensively investigated (Keyes, 2005), and many pathological outcomes were found. With the advent of positive psychology in the past few decades, a shifted focus on well-being or overall happiness was more embraced in the hope of mobilizing an individual’s internal capacity or strength to achieve people’s best performance. Or rather, well-being is regarded as an integral human pursuit (Tang and Wang, 2020). Eudemonic well-being (EWB) is one of the main concepts accounting for happiness with a focus on the meaning of life and self-development (Dolan and Metcalfe, 2012). An understanding of their EWB is crucial, particularly after their recent volunteer experience. This is not only for the good of their own psychological balance, but also for more sustainable development of volunteer organizations, which normally suffer from a shortage of talents, wherein “it is generally much easier for volunteers to quit their volunteer employer than it is for salaried workers to quit theirs” (Alfes et al., 2016, p. 596).

Well-being and “authentic happiness” can be achieved through being absorbed into activities and meaning-making tasks, as posited in Seligman (2011) PERMA block building theory (Seligman, 2011). In other words, proper involvement in meaningful cause is believed to enhance people’s well-being. Controversially, it was identified that improper involvement, such as over-involvement, was found to be detrimental, thus leading to many negative outcomes in the workplace and personal life (Claxton et al., 2017; Edmondson et al., 2019). Risky volunteer service can elevate some mental health issues (Milligan-Saville et al., 2018). Regretfully, rare empirical studies could be found so far about whether the meaning-making task in the context of volunteering in general, or public emergency crisis in particular, can contribute to volunteers’ EWB. To fight against possible adverse circumstances, empathy was identified to benefit people by allowing them to forgo some unpleasant experiences and offset the challenging stress (Muralidharan and Kim, 2020), thus mitigating some psychological reactance (Shen, 2010). As a valuable human quality in volunteerism (Butler and Tomazos, 2011), empathy was tested as a moderator that can regulate some behavioral variables and emotional responses or valuable orientation in different contexts (e.g., Lindsey et al., 2007). Despite the urgency and the importance of better understanding the ways volunteers reenergize or invigorate themselves into a challenging work environment through involvement, prior academic research remains sparse about its exerting influence on their EWB moderated by empathy.

To address these gaps, this study aims to measure volunteers’ EWB after they finish their recent volunteer job in a public health emergency, soliciting volunteers’ responses after their volunteering services in Wuhan of China during the first phase of the pandemic outbreak. We further seek to identify volunteers’ behavioral attitudes, subjective norms, and perceived control drawing on the Theory of Planned Behavior (TPB; Ajzen, 1991) and investigate their respective relationships with EWB. What is more, the moderating role of empathy between volunteers’ job involvement and EWB in public health emergencies got substantiated.

## Theoretical Background and Hypothesis

### Eudemonic Well-Being

Eudemonia was conceptualized as personal expressiveness to represent happiness along with hedonic enjoyment. When individuals strive to live based on the notion of their “true” selves, and when they realize their potential (self-actualization), “personal expressiveness” will occur. In other words, happiness occurs when people engage in activities that best match their deep-seated values and in which they could invest wholeheartedly (Waterman, 1993).

Although the measurement of EWB was described to be “less strictly defined” (Vanhoutte, 2014, p. 5) compared to other well-being concepts, such as hedonic, still, as summarized by Vanhoutte (2014), there are mainly three different measurement approaches. One is to conceptualize it into six dimensions by Ryff and Keyes (1995), which include autonomy, environmental mastery, personal growth, positive relations with others, purpose in life, and self-acceptance. The second is underpinned by self-determination theory with a focus on three dimensions: autonomy, competence, and relatedness (Ryan and Deci, 2000). The third is often short for CASP-19 (Hyde et al., 2003), which consists of control, autonomy, self-realization, and pleasure. Among them, the first approach is deemed relatively more suitable in the context of the volunteer domain given the following reasons.

First, although some common or similar dimensions were shared, a distinction can still be spotted. The dimension of pleasure included in the third approach seems not to be appropriate in a public health emergency like the COVID-19 pandemic. Furthermore, life purpose and environmental mastery are only presented in the first approach, which fit right into the mechanism of enhancing well-being in volunteerism. The feeling of making a difference by doing altruistic things triggers positive well-being (Piliavin and Siegl, 2007). Herein, life purpose is a vital dimension for this study. Environmental mastery was defined as an individual’s ability to “control and manage one’s environment” (Hill and Allemand, 2010, p. 246). Confronting the challenging and critical environment on a voluntary basis brings up rare and valuable qualities. Besides, the first approach of six-dimension measurement was previously adopted and tested empirically in volunteer-related studies (e.g., Son and Wilson, 2012). Based on the aforementioned findings, the first approach of dimensionality is deemed appropriate for this study.

### Theory of Planned Behavior and Well-Being

Ajzen’s (1991) TPB is an established behavioral theory for exploring determinants accounting for an individual’s behavior intentions based on a premise that decision-making is rational (Ajzen and Fishbein, 1980). The theory introduces three variables, including behavioral attitudes (AT), subjective norms (SN), and perceived control (PC). AT is related to a person’s evaluation of the performance of certain behavior. SN refer to the perceived or actual social pressure exerted by others, normally coming from people with less social distance known as “significant others,” such as family members, teachers, industrial managers, and other important reference figures in the decision-making process (Ajzen, 1991; Goh and Lee, 2018). PC is associated with the perception of the difficulty or ease of performing certain behavior (Ajzen, 1991).

Theory of Planned Behavior is widely adopted in examining various behaviors in the social science domain, including volunteerism, and is empirically identified as a more viable tool in explaining volunteers’ participation with better predictive validity than other approaches (Greenslade and White, 2005). People’s attitude toward a particular event predicts their personal growth and purpose in life (Lo et al., 2016). To comply with important referents is a normative belief. For volunteers, being seen as a “hero” (Tomazos and Butler, 2010, p. 363) in many situations represents an honor, which may further strengthen their EWB. As a cognitive psychological resource (Bandura, 2001), an individual’s perceived control can decrease personal stress (Cohen, 1980), so as to avoid harm to mental health. It was found to buffer the negative effects of accumulative stress by transforming to subjective well-being (SWB) (Jibeen, 2019).

Unlike many previous studies that focus on participants’ intentions as the outcome variables in TPB theory, we argue for the value of their EWB after their recent volunteer experience in the emergency event. We seek to understand the antecedent factors that induced them to volunteer in a public health emergency. Given this, the following hypotheses were proposed for volunteers who participated in a public health emergency:

H1a: Volunteers’ attitude has a significant and positive effect on their EWB after their completion of volunteer participation.

H1b: Volunteers’ subjective norms have a significant and positive effect on their EWB after their completion of volunteer participation.

H1c: Volunteers’ perceived control has a significant and positive effect on their EWB after their completion of volunteer participation.

### Job Involvement and Well-Being

Involvement is defined as the extent to which an individual can internalize values of goodness or the importance of work (Lodahl and Kejnar, 1965), which is manifested by the amount of physical and psychological energy devoted to the job (Wolf-Wendel et al., 2009). Job involvement was first proposed by Allport (1943) as a type of job attitude, but later on, Kanungo (1982) definition dominated the discourse of the literature (Salessi and Omar, 2019), with a focus on the degree to which individual values and identifies his job. This term is sometimes used interchangeably with engagement (Hallberg and Schaufeli, 2006), with a common emphasis on the level of absorption and engrossment. In this study, it refers to the degree to which volunteer participants value and identify their volunteer tasks during the emergency event.

The positive relationship between job involvement and well-being has been extensively substantiated by researchers (e.g., Srivastava and Krishna, 1992; Srivastava, 2001). Besides, it receives solid support from Seligman (2011) PERMA block building theory, which asserts that well-being and “authentic happiness” can be achieved through being absorbed into activities and meaning-making tasks. However, the relationship between involvement and enhanced well-being remains ambiguous, with very few studies rendering direct empirical support in the context of volunteerism. On one hand, the adversities and challenging difficulties in public health emergencies might confound or complicate this relationship. On the other hand, the degree of involvement in this context may further add complications to this relationship. For instance, over-involvement in the workplace was found to suppress the mental health in an elementary school (Gechman and Wiener, 1975). This finding was somewhat different from that of Riipinen (1997), who stated that job involvement and well-being are conditioned by need fulfillment. Similarly, an indirect and positive relationship was also identified between job involvement and SWB through the experience of flow in work (Zhou et al., 2019).

Lodahl and Kejnar (1965) job involvement scale has been widely acknowledged. Subsequent adaptations were made afterward (Kanungo, 1982; Reeve and Smith, 2001). A 10-item Job Involvement Questionnaire (JIQ) was developed and validated as a simple version (Kanungo, 1982), exhibiting high internal consistency, validity, and reliability (Paterson and O’ Driscoll, 1990). Moreover, this JIQ is suggested to be suitable to measure psychological identification at the individual level (Igbaria et al., 1994). Thus this measurement was adopted by our study.

Based on the above-mentioned discussion, we propose the following hypothesis:

H2: Volunteer’s job involvement has a significant and positive effect on their EWB after their completion of volunteer participation in a public health emergency.

### Empathy

Empathy plays a vital role in contributing to ethical life, moral judgment, and prosocial behavior (Eisenberg, 2000). The interdisciplinary academic effort, ranging from neuroscience, psychotherapy, psychology, and philosophy, has been made, viewing empathy as a kind of phenomenon, emotional reaction, trait, or capability. Empathy was normally seen as a vicarious response to another person’s emotional state or condition (Eisenberg and Fabes, 1990). It was characterized by being able to resonate with another person’s mind of state. Many researchers believe that empathy is an interaction between both facets, that is, emotional and cognitive (e.g., Coke et al., 1978). In a similar vein, Stueber (2013) contends that there are two striking features of empathy: ability to recognize other people’s feelings and ability to engage with others.

Stemming from the very nature of caring for other people in a social relationship, empathy has been employed to understand various helping behaviors to maintain social justice, promote well-being, and facilitate altruistic behavior. In addition, it has often been entangled and interchangeably used with other similar terms, such as sympathy or compassion (e.g., Eisenberg and Miller, 1987). Nevertheless, it is pointed out that sympathy was tapped by one of the empathy’s sub-scales, namely, emphatic concern (Davis, 1983). When filled with empathy, people’s empathic reaction will lead to two actions: one is compassion which is categorized by other related good feelings such as warmth and love, and the other is empathic distress which taps self-related negative feelings such as personal stress (Singer and Klimecki, 2014). Given this, empathy is employed as an umbrella term in this study.

In recognizing its full-fledged theoretical implication, a multi-dimensional approach is mainly embraced by studies in philosophy and behavioral science instead of a unipolar one. Among many options, Davis (1983) Interpersonal Reactivity Index (IRI) was identified as a valid and reliable instrument (e.g., Pulos et al., 2004), which taps the two important facets in a comprehensive fashion with four sub-scales, including perspective taking (PT), fantasy (FS), empathic concern (EC), and personal distress (PD). This IRI instrument remains rigorous when getting tested in a cross-cultural context (Karyagina and Kukhtova, 2016). As suggested, each sub-scale of IRI is a separate facet of empathy and can be singled out to be independently used (Thomas et al., 2007). This study adopts PT and PD to capture volunteers’ cognitive and emotional facets. As the only cognitive facet, PT is seen as a core component of empathy (Davis, 2018), which plays a positive role in enhancing well-being (Gleichgerrcht and Decety, 2013). Another study confirmed the interaction effect of PT with human responsibility in predicting the positive helping of a victim (Marjanovic et al., 2012). As a component of emotional empathy, PD taps the negative effect of the exposure to disaster (Heath et al., 2020). This is deemed essential to unpack the mental state after the completion of a stressful emergency task. The moderating role of PD was confirmed among volunteers in a prior study, revealing that low PD is beneficial for volunteers’ emotional relief in disaster aftermath (Cristea et al., 2014). Based on this finding, this study proposes the following hypotheses:

H3a: Volunteers’ PT significantly and positively moderates the effect of job involvement on their EWB. The effect is stronger when PT is high compared to when PT is low.

H3b: Volunteers’ PD significantly and negatively moderates the effect of job involvement on their EWB. The effect is weaker when PD is high compared to when PD is low.

**FIGURE 1:**
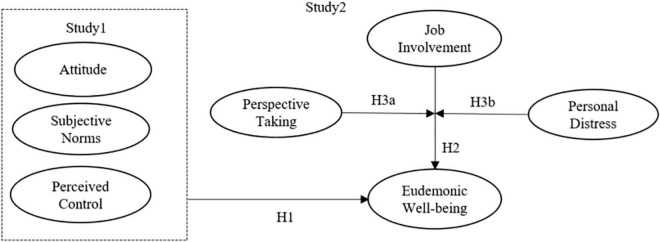
Research model. Source: Authors.

The research model is shown in Figure 1.

## Method

A sequential exploratory strategy of mixed methodology was employed with a research design of qualitative study followed by a quantitative one. The qualitative study aims to identify volunteers’ attitudes, social norms, and perceived control drawing upon the TPB from an in-depth interview. The subsequent quantitative study further contributes to testing the influence of the above-mentioned variables on the EWB of volunteers. This strategy can help identify the “elements of a theory” and extend the horizon by investigating the relative importance of these elements in improving the EWB of volunteers’, with authors investing great effort in overcoming the strategy’s “very time-consuming” nature (Terrell, 2012, p. 264). Furthermore, a nested method was adopted, with the identified attitudes, social norms, and perceived control embedded in the proposed normative framework, and further exploring their relationship with EWB. In this nested design, job involvement was integrated, accentuating its vital significance in internalizing the value of morality in expressing themselves in volunteering. Both the interview and survey were conducted within 1 month of the completion of their volunteer work in COVID-19, to ensure the recollection of their recent memory remained relatively fresh and clear.

## Study 1: Qualitative Data from Interviews

### Sampling and Interview Questions

A snowball sampling method (SSM) was used in our quantitative study. As a non-probability convenience technique, it is used when potential subjects are not easily identifiable (Petersen and Valdez, 2005) or when the research timeline needs to be efficient. In the initial stage, we reached five volunteers who were, respectively, from Shandong Province and Jiangsu Province medical assistance teams, the Wuhan hospitality industry, and the Sichuan volunteer group in China. Each of these organizations was established long enough and comprised experienced volunteers. They introduced the other 20 qualified volunteers to this study. Totally, we selected 25 experienced volunteers. As can be seen from Table 1, the respondents included 25 volunteers, covering job positions ranging from frontline health workers (5), transportation workers (2), vehicle convoy drivers (2), isolation area staff (2), catering and hospitality staff (7), community workers (4), to online psychological assistants (3), and all of them volunteered during the COVID-19 public health emergency. Most of them were between 21 and 35 years of age (68%). The total number of 25 volunteers met the sample size recommended by many TPB-related studies (*N* ≥ 25) (e.g., Ajzen and Fishbein, 1980; Godin and Kok, 1996; Goh and Lee, 2018).

**TABLE 1:** Personal profiles of interviewees in study 1 (*N* = 25).

	Description	No.	%
Types of volunteer job	Front-line health worker	5	20%
	Transportation worker	2	8%
	Vehicle convoy worker	2	8%
	Catering worker	3	12%
	Hospitality worker	4	16%
	Isolation area worker	2	8%
	Community worker	4	16%
	Psychological assistance worker	3	12%
Gender	Male	11	44%
	Female	14	56%
Age	21–25	2	8%
	26–30	6	24%
	31–35	9	36%
	36–40	4	16%
	41–45	4	16%

The interviews were conducted after obtaining informed consent. All interviews lasted for an average of 30 min. Online audio interviews were recorded with all the respondents to avoid direct human contact, as a precautionary measure due to the epidemic situation. All authors made joint efforts in transcribing and coding jobs to ensure accuracy and reliability. All interviews occurred from 10 April 2020 to 10 May 2020. Three interview questions were developed based on TPB (details of the interview questions included in study 1 are given in Appendix). Respondents were asked to identify their attitudes, list the influential social group, and outline any possible perceived difficulties in doing their volunteer jobs during the public health emergency, and how they dealt with those difficulties. Content analysis was conducted to extract possible themes with inductive techniques (Boyatzis, 1998). An initial pool consisting of 13 themes was generated under these three variables. We further invited two subject experts to go through the script and themes to ensure content validity. The finalized versions were adjusted to 12 themes, by combining “being away from family or friends” and “keeping secrets from family or friends” into one theme of “shunning of family/friends.”

### Findings

Eight AT, eight SN, and nine PC themes were identified from the 25 respondents. The top four items for AT were altruism (72%), fulfilling one’s ambition (64%), mentally and emotionally exhausting (56%), and a sense of helplessness (52%). The top four items for SM were frontline health workers (80%), family and friends (52%), other volunteers (52%), and government/community/volunteer organizations (52%). The top four items for PC were high risk of infection (80%), shortage of supplies for epidemic prevention (72%), lack of effective medicine/therapeutic schedule (56%), and shunning of family/friends (56%). The findings of study 1 are summarized in Table 2.

**TABLE 2:** The results of study 1, antecedent factors of TPB.

		Frequency (percentage)	Examples of selected interview transcript
AT1	Altruism	18 (72%)	Helping front-line medical staffs to relieve psychological stress. (V10)
AT2	Fulfilling one’s ambition	16 (64%)	Saving lives, that is why I chose to go into medicine. (V2)
AT3	Mentally and emotionally exhausting	14 (56%)	Sometimes I do really tired, very worry about the supplies late delivery even overtime every day. (V4)
AT4	Sense of helplessness	13 (52%)	There were not enough supplies, staffs, beds and experience at the beginning, I remember I was so anxious to cried once time. (V1)
SN1	Frontline health workers	20 (80%)	Front-line health workers are more laborious than us. (V11)
SN2	Family and friends	13 (52%)	My father was a front-line health work during SARS, he is my hero and I also want to learn from him. (V3)
SN3	Other volunteers	13 (52%)	I have received the help from other volunteers when my hometown was affected by the earthquake in 2008. (V5)
SN4	Government/community/volunteer organization	13 (52%)	I saw the mobilization from the volunteer organization, I think I have to do something. (V20)
PC1	High risk of infection	20 (80%)	My responsibility was food delivery and collection in the isolation area, and I was feared of infection especially when tidy up the leftover food. (V15)
PC2	Shortage of supplies for epidemic prevention	18 (72%)	There were not enough disinfection protective materials in the community at the beginning. Finally, we received emergency masks and disinfectant liquid after contacting with many departments (V20).
PC3	Lack of effective medicine/therapeutic schedule	14 (56%)	Once time I saw the exacerbation of patients, but I haven’t a better therapeutic to control it that time. (V22)
PC4	Shunning of family/friends	14 (56%)	I just told my mother I was send out to study, because she is not very well and I’m afraid she will too worried about me. (V3) I contact with suspected case during work, so I must isolation from my families. (V9)

*AT, attitude; SN, subjective norms; PC, perceived control.*

## Study 2: Quantitative Analysis

### Sampling and Measures

Interviewees in study 1 were further asked to share the questionnaire link with their volunteer organizations, asking for their cooperation to distribute the link to as many as possible volunteers who participated in a recent public health emergency. Interviewees in study 1 were not included in study 2. The snowball sampling method was used, which was deemed appropriate to ensure efficient access to representative samples during the COVID-19 epidemic while social distancing was strictly enforced. The questionnaires were distributed and collected from 13 May 2020 to 20 June 2020. According to the normal distribution:


(1) $$n={\left[\frac{e\,r\,f^{-1}\,\left(C\right)}{\sqrt{2}\times w}\right]}^{2}$$

From WolframAlpha (2022)^1^, *erf*^−1^(0.95)*was* 1.38590, when confidence level (*C*) was 95% and confidence interval (*w*) was 5%. A minimal size (*n*) of about 384 respondents was needed for our research. Of the 411 questionnaires collected in study 2, 392 were found to be valid; thus, the effective rate was 95%.

The questionnaire consisted of five sections: TPB antecedent factors, JI, empathy, EWB, and demographic information, such as gender, age, and type of volunteering. The original question items were developed in English and translated into Chinese following the back-translation procedure (Brislin, 1980). The four main parts of the questionnaire were measured on a seven-point Likert scale (1 = strongly disagree, 7 = strongly agree). Negative items were scored in a reverse pattern. SPSS 23.0 and PROCESS macro v3.3 were used for data analysis. PROCESS macro can process traditional regression functions with interaction going “automatically and painless,” while yielding “substantively identical” results (Hayes et al., 2017, pp. 80–81). Furthermore, it is labeled as a “versatile modeling tool,” as it is capable of integrating multiple functions (Hayes, 2018). Besides, the PROCESS method determines the significance of interaction terms and calculates the degree of the moderating effect in high-level and low-level groups with a simple slope in a more readable manner. Template Model 1 in PROCESS, which illustrates a moderation relationship template, was adopted for this study.

The high-frequency (more than half) items that can present the opinion of the majority of participants of study 1 (Goh, 2009; Goh and Ritchie, 2011; Goh and Lee, 2018) were used to design questionnaire items in study 2. Regarding the attitude, typical statements were adopted, such as “overall my experience, this volunteering for this public health emergency was an altruistic behavior.” For the SN item, the typical statement was, “most people (frontline health workers, family and friends, etc.) who are important to me would encourage me to be volunteers in this public health emergency.” A typical statement for the measurement of PC was “I’ve control over being away or keeping secret from family and friends.” The JIQ (Kanungo, 1982) was adopted to measure the respondents’ volunteer job involvement. Six dimensions, including autonomy, environmental mastery, personal growth, positive relations with others, purpose in life, and self-acceptance, were used to evaluate the respondents’ EWB (Ryff, 1989; Ryff and Singer, 2008; Son and Wilson, 2012). The cognitive and emotional dimensions of the IRI, namely PT and PD, were used to measure empathy (Davis, 1983).

### Personal Profile, Reliability, and Validity

Of all the respondents, 53.3% were men and 64.8% were young people (aged 18 to 30 years). As seen in Table 3, the Cronbach’s alpha of all the included constructs ranges between 0.776 and 0.973, exhibiting satisfactory reliability. The Kaiser–Meyer–Olkin measure of sampling adequacy (KMO) and Bartlett’s test of sphericity were used to test construct validity. In this study, most KMO values were between 0.601 and 0.955, except for PT and PD which had a value of 0.500, which is probably due to the fact that both these two variables included only two items. The results show that the proposed scales indicated acceptable construct validity (Field, 2005). The above-mentioned relevant information is detailed in Tables 3, 4.

**TABLE 3:** Descriptive statistics of study 2 (*N* = 392).

		Frequency	Percentage
Gender	Female	183	46.68%
	Male	209	53.32%
Age	1 = Below 18	5	1.28%
	51∼60	15	3.83%
	41∼50	37	9.44%
	26∼30	105	26.79%
	18∼25	110	28.06%
	31∼40	120	30.61%
Type of volunteering task	Construction worker	25	6.38%
	Vehicle convoy worker	30	7.65%
	Front-line health worker	60	15.31%
	Transportation worker	66	16.84%
	Community worker	115	29.34%
	Hospitality worker	96	24.49%

**TABLE 4:** Questionnaire items, reliability, validity, mean scores, and standard deviations of study 2.

Dimension		Item
**TPB**		
AT1	Attitude (mean = 5.897; SD = 0.445)	Altruism
AT2		Fulfilling one’s ambition
AT3		Mentally and emotionally exhausting
AT4		Sense of helplessness
SN1	Subjective norms (mean = 5.872; SD = 0.427)	Frontline health workers
SN2		Family and friends
SN3		The other volunteers
SN4		Government/community/volunteer organization
PC1	Perceived control (mean = 5.929; SD = 0.573)	High risk of infection
PC2		Shortage of supplies for epidemic prevention
PC3		Lack of effective medicine/therapeutic schedule
PC4		Being away or keeping secret from family and friends
**Job involvement** (mean = 6.083; SD = 0.573)		
JI1	The most important things that happen to me involve my volunteering job	
JI2	To me, my volunteering job is only a small part of who I am	
JI3	I am very much involved personally in my volunteering job	
JI4	I live, eat and breathe my volunteering job	
JI5	Most of my interests are centered around my volunteering job	
JI6	I have very strong ties with my volunteering job which would be very difficult to break	
JI7	Usually, I feel detached from my volunteering job	
JI8	Most of my personal life goals are volunteering job-oriented	
JI9	I consider my volunteering job to be very central to my existence	
JI10	I like to be absorbed in my volunteering job most of the time	
**Empathy** (mean = 5.292; SD = 0.876)		
PT1	Perspective taking (mean = 6.042; SD = 0.375)	I try to look at everybody’s side of a disagreement for I make a decision.
PT2		I believe that there are two sides to every question and try to look at them both.
PD1	Personal distress (mean = 4.541; SD = 1.684)	In emergency situations, I feel apprehensive and ill-at-ease.
PD2		When I see someone who badly needs assistance in an emergency, I go to collapse.
**Eudemonic well-being** (mean = 6.436; SD = 0.451)		
SA1	Self-acceptance (mean = 6.393; SD = 0.488)	Pleased with how things turned out so far
SA2		Satisfied with my achievements
PR1	Positive relations with others (mean = 6.462; SD = 0.484)	Giving person, sharing time with others
PR2		Experienced many warm and trusting relations
PG1	Personal growth (mean = 6.426; SD = 0.490)	Challenging new experiences are important
PG2		Life has been continuous process of growth
PL1	Purpose in life (mean = 6.550; SD = 0.471)	I think life has a clearer direction
PL2		I am not wander aimlessly
EnM1	Environmental mastery (mean = 6.390; SD = 0.496)	Good at managing responsibilities of daily life
EnM2		Feel I am in charge of situation in which I live
AM1	Autonomy (mean = 6.392; SD = 0.501)	I have autonomy with my work
AM2		I am confident with my own opinions

*N = 392.*

*Resource: Davis (1983); Ryff (1989); Canniff (1990); Ajzen (1991); Son and Wilson (2012).*

### Hypothesis Testing

#### Regression Analysis

We used linear regression analysis to identify the influence and extent of the influence of TPB antecedents and JI on EWB. Gender and age were computed as control variables.

Three regression models (Models 1, 2, and 3) are shown in Table 5 with EWB as outcome variables. Model 1 is significant (*F* = 18.257, *p* < 0.001, *R*^2^ = 0.191). The independent variables in Model 2 include AT, PC, and JI, with the significant outcomes (*F* = 85.685, *p* < 0.001) and higher explanatory power than that of Model 1 (*R*^2^ = 0.642). AT has a significant positive impact on EWB (β = 0.570, *p* < 0.01), as does PC (β = 0.488, *p* < 0.01). The impact of SN on EWB is not significant. Thus, Hypotheses 1a and 1c are supported, while Hypothesis 1b is not. Model 3 yields satisfactory explanatory power (*R*^2^ = 0.490), with JI serving as a significant independent variable, which indicates JI has a significant positive influence on EWB (β = 0.766, *p* < 0.001). Thus, Hypothesis 2 is supported.

**TABLE 5:** Results of regression analysis and moderating effective test.

Model 1		Model 2		Model 3		Model 4		Model 5	
Beta	*t*	Beta	*t*	Beta	*t*	Beta	*t*	Beta	*t*
−0.103	−2.207*	−0.228	−7.003**	−0.070	−1.883	−0.045	−1.349	−0.043	−1.565
−0.006	−0.127	0.182	5.226**	0.280	6.524***	0.121	7.148***	0.097	7.285***
\	\	0.570	2.947**	\	\	\	\	\	\
\	\	−0.039	−0.198	\	\	\	\	\	\
\	\	0.488	8.665**	\	\	\	\	\	\
\	\	\	\	0.766	15.015***	0.672	15.863***	0.478	14.192***
\	\	\	\	\	\	−0.201	−4.341***	\	\
\	\	\	\	\	\	0.164	2.630*	\	\
\	\	\	\	\	\	\	\	−0.159	−16.160***
\	\	\	\	\	\	\	\	−0.052	−3.088**
18.257***		85.685***		61.638***		51.367***		111.913***	
0.191		0.642		0.490		0.518		0.700	

N = 392, ***p < 0.001, **p < 0.01, *p < 0.05.

*Outcome variable: EWB.*

*All data were non-standardized in moderating effect test.*

*AT, attitude; SN, subjective norms; PC, perceived control, JI, job involvement; PT, perspective taking; PD, personal distress.*

#### Moderating Effect Test

The results of moderating models (Model 4 and Model 5) in Table 5 show that both PT and PD exert a significant moderating effect on the relationship between JI and EWB (β_JI*PT_ = 0.164, *p* < 0.05; β_JI*PD_ = −0.052, *p* < 0.01).

We further tested these moderating effects on different levels, respectively, as shown in Table 6. Both a low PT and a high PT can positively moderate the positive effects of JI on EWB [SE_*PT(M–1SD)*_ = 0.611, t_*PT(M–1SD)*_ = 12.612, *p* < 0.001; SE_PT(M+1SD)_ = 0.734, t_*PT(M*+1SD)_ = 15.163, *p* < 0.001]. A similar finding was found with PD [SE_*PD(M–1SD)*_ = 0.566, t_*PD(M–1SD)*_ = 12.164, *p* < 0.001; SE_PD(M+1SD)_ = 0.402, t_PD(M+1SD)_ = 10.215, *p* < 0.001]. This result suggests that the positive effect of JI on EWB is positively moderated by both PT and PD. The results provide full support to Hypothesis H3a but partial support to Hypothesis H3b.

**TABLE 6:** Moderating effects of PT and PD on different levels.

Variable		Conditional effects of the focal predictor at values of the moderator				
		Effect	SE	LLCI	ULCI	t
PT	M-1SD	−0.375	0.611	0.515	0.706	12.612***
	M	0	0.672	0.589	0.755	15.863***
	M+1SD	0.375	0.734	0.638	0.829	15.163***
PD	M-1SD	−1.684	0.566	0.475	0.658	12.164***
	M	0	0.478	0.412	0.545	14.192***
	M+1SD	1.459	0.402	0.325	0.48	10.215***

*** p < 0.001.

*PT, perspective taking; PD, personal distress.*

**FIGURE 2:**
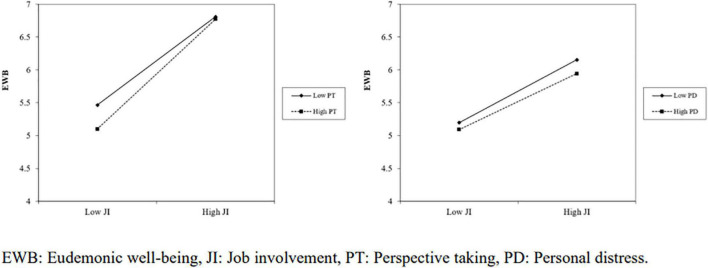
Simple slope plots of interactions.

The simple slope plots (Figure 2) further reflect the degree of these moderating effects. A high PT positively moderates the influence of JI on EWB, whereas a weak moderating effect relaxes the same relationship. However, a high moderating effect of PD reduces the positive influence of JI on EWB when compared to a low moderating effect of PD. Thus, it can be seen that the positive effect of JI on EWB is conditional and dependent on the moderating effects of both PT and PD.

## General Discussion

The contribution of this study is triple faceted. First, it extends the existing literature on volunteerism by particularly identifying the EWB of volunteers after their completion of volunteer tasks in the context of a public health emergency, seeking a better vision of their transformed life goals and personal growth from the perspectives of EWB. The completion of a volunteer task is likely to bring out a “turning point” (Starr, 1994, p. 137) or even “rebirth” (Tomazos and Butler, 2010, p. 363) for most volunteers. The disruption of life due to COVID-19 could last for a considerably longer period of time pervasive in many domains of human life, with a rising need for volunteers in a similar context. Second, a mixed method was designed in this study. This study is unique in that it used content analysis to generate a questionnaire that targeted the planned behaviors of volunteers. With a sequential and nested approach, this mixed study is expected to generate more insights into the mental state of volunteers (Chen and Chen, 2011). Volunteers’ dedication toward their job, “the significant others” who exerted social pressure on them, and volunteers’ perception of the difficulty were outlined in this research, together with the respective relationship with their EWB. This broadened understanding might serve as one of the valuable endeavors to unpack this group of unpaid workforces by exploring how they cope with pandemic-related uncertainties and challenges. Last but not least, this research also considers the role of empathy in the context of volunteerism in general, highlighting the regulating role of PT and PD in predicting volunteers’ EWB. So far, only a limited number of studies addressed the role of empathy as a moderator. This study joins in this limited stream of research by adopting this perspective and extending empathy to volunteerism in general and to volunteerism during the pandemic crisis in particular.

The high-frequency items from study 1 were extracted and framed into study 2. The list of attitudes that guide volunteer participation was generated, including altruism, fulfillment of ambitions, mental and emotional exhaustion, and a sense of helplessness. It can be seen that the volunteers mainly embrace other-oriented values to help and serve people in crisis, as altruism was listed as the most favorable appraisal of volunteerism. Fulfilling ambitions ranked second, which supports the argument that volunteering motives can be seen as a continuum between self-oriented and other-oriented values (Curtin and Brown, 2019), or between intrinsic and extrinsic orientation (Finkelstien, 2009). Volunteers’ attitude toward volunteerism can positively boost their EWB when completing their tasks, including even negative attitudes. It may be inferred that when volunteers take a retrospective look back on their journey, to be able to navigate and get over all the hardship presents a milestone or breakthrough for personal growth.

In terms of SN, frontline health workers, family and friends, other volunteers, and government/community/volunteer organizations were the top four sources of social pressure as revealed in study 1. In the public health emergency environment of the COVID-19 epidemic, the frontline health workers serve as the strongest reference group for volunteers according to study 1. It might be their dedication that highly motivates their peers. Volunteers respond actively to them, which indicates that there is still plenty of sizable room for volunteer organizations and governments to better engage with them and facilitate efficient volunteer recruitment. It is important to note that although volunteers are inclined to comply with these referents’ beliefs to gain possible approval, SN shows no direct influence on volunteers’ EWB in study 2. This is probably because people who participate in volunteering behavior tend to have high internal control and self-efficiency (Lee, 2018). It might be particularly the case for volunteers in public health emergency who normally have great willpower and perseverance to face the challenges.

Regarding PC, the high risk of infection, shortage of supplies for epidemic prevention, lack of effective medicine/therapeutic schedule, and being away or keeping secret from family/friends were perceived as the most serious difficulties by volunteers in study 1. This perception of control of beliefs can significantly contribute to volunteers’ EWB. As contended by Ajzen (1991), the controlling factors may be categorized as an internal one, which is related to self-efficacy and knowledge, and an external one, which is related to the environment. As coronavirus is highly contagious, some precautionary measurements, such as distribution of PPE and rationalizing the donated materials, can effectively ease the perceived difficulties and barriers during the task, consequently boosting the EWB of volunteers. Thus, volunteers’ mental health should be monitored regularly, and effective interventions need to be made available on a regular basis, not only before or during, but also at the post-pandemic stage.

Among all the dimensions of EWB, purpose in life and personal growth ranked at the top, with mean values of 6.550 and 6.462, respectively. It can be inferred that respondents have a fairly satisfactory level of EWB after completing their volunteering activities during the pandemic. This confirms our hypothesis that the effects of attitude and PC on volunteers’ EWB were significantly positive, while the impact of SN was not. Although SN offer guidance from references for volunteers, they do not directly influence EWB. JI has a significant positive effect on the volunteers’ EWB. The studies of Riipinen (1997) and Zhou et al. (2019) helped in shedding light on this finding. JI has a positive impact on the well-being of volunteers when they are fulfilled through sentiments of growth and development, which may further help ameliorate the psychological burdens of volunteers.

Although both PT and PD exhibit moderating effect on the influence of JI on EWB, their modulating power is inconsistent at different levels. Volunteers with a higher level of PT are more likely to experience better EWB than those with a lower level of PT. However, volunteers with a higher level of PD are less likely to experience positive EWB compared to those with a lower level of PD. Enhancing volunteers’ PT skills presents an effective strategy to help volunteers, particularly in public health emergency environment which needs a quick response or a large volume of assistance. It probably results from the fact that when volunteers have sensitive insight into other peoples’ feelings and thoughts, it is easier to engage in problem-solving activities more efficiently. This result is consistent with the research of López-Pérez (2013), which reports that professional health workers with higher PT enjoy better performance. As an affective dimension of empathy, lower PD is beneficial for the stress relief of volunteers in disaster aftermath (Cristea et al., 2014). Hence, governments and volunteer organizations should take precautions to channel their distress or regulate it within a reasonable capacity.

Positive JI is likely to exert a more positive EWB among volunteers with high PT compared to volunteers with a low PT during COVID-19 volunteering work. This finding denotes that volunteers with a high PT can benefit from a stronger relationship between JI and EWB. When the volunteers have the ability to look beyond themselves and extend their perspectives on other people, in other words, putting themselves in the recipients’ shoes and considering problems from multiple aspects, they are more likely to gain personal growth and find the purpose of their task. As an important component of affective empathy, PD does not appear to exert negative effects all the time as we previously assumed. A moderate level of PD can motivate them to be as adaptable as possible to a stressful environment to tap their potential. However, volunteers with a high PD would not enjoy that situation. It might be inferred that only the right amount of PD can help volunteers gain self-actualization.

Although efforts were made to diversify job types and demographics of volunteers for the purpose of sampling, the study could not be seen as entirely free of bias. In the future, broadening the volunteer job types, recruiting a larger sample, and comparing volunteers’ EWB among various geographic and cultural contexts warrant further academic attention. Certain factors, such as age cohort, educational level, income factor, personal health, and honor incentives, may also influence volunteers’ EWB in emergency events, and thus is worthy of further investigation. Besides, although this study did not aim to explore volunteers’ intention to participate further in a similar volunteering task in a near future, which was out of the scope of this study, longitudinal research is suggested in a middle-range or even long term for volunteers who ever participated in this unprecedented public health emergency rescue with their mental state and subsequent volunteering behavior.

## Data Availability Statement

The original contributions presented in the study are included in the article/supplementary material, further inquiries can be directed to the corresponding author.

## Author Contributions

JT, X-CL, and XZ were involved in the conceptualization, literature review, methodological design, investigation, data analysis, writing, and review. All authors have read and agreed to the published version of the manuscript.

## Conflict of Interest

The authors declare that the research was conducted in the absence of any commercial or financial relationships that could be construed as a potential conflict of interest.

## Publisher’s Note

All claims expressed in this article are solely those of the authors and do not necessarily represent those of their affiliated organizations, or those of the publisher, the editors and the reviewers. Any product that may be evaluated in this article, or claim that may be made by its manufacturer, is not guaranteed or endorsed by the publisher.

## Footnotes

1
http://wolframalpha.com/

## Appendix: Interview Questions

The Appendix comprises the interview questions included in study 1:

Question 1: What are the attitudes of volunteers toward volunteering behavior? (Positive/negative).

Question 2: Who are the important people/social groups that influence volunteers toward volunteering behavior?

Question 3: What are the perceived difficulties/barriers faced by volunteers toward volunteering behavior?

**APPENDIX TABLE A1:**

	Gender	Age	Type
1	F	21–25	Health worker
2	F	36–40	Health worker
3	M	31–35	Health worker
4	M	36–40	Transportation
5	F	26–30	Food and beverage
6	F	26–30	Hospitality
7	M	21–25	Hospitality
8	F	31–35	Community management
9	F	31–35	Transportation
10	M	41–45	Psychological assistance (for health worker)
11	F	41–45	Psychological assistance (online for public)
12	M	31–35	Transportation
13	F	31–35	Health worker
14	M	21–25	Construction
15	M	21–25	Transportation
16	F	26–30	Food and beverage
17	F	21–25	Health worker
18	M	26–30	Food and beverage
19	F	21–25	Health worker
20	F	31–35	Health worker
21	M	36–40	Transportation
22	M	26–30	Transportation
23	F	31–35	Hospitality
24	F	41–45	Hospitality
25	F	36–40	Hospitality
